# Supplementary material for: COMPAR-EU Recommendations on Self-Management Interventions in Type 2 Diabetes Mellitus
Source: Healthcare (Basel). 2024 Feb 16;12(4):483. doi: 10.3390/healthcare12040483 (PMC10887949; doi:10.3390/healthcare12040483)
Supplement: Supplementary file 1 [file healthcare-12-00483-s001.zip › healthcare-2708802-supplementary.pdf]

Supplementary Table S1: Self-management interventions' components description

Component Abbreviation	Component description	Strategies included
**AB**	Action-based behavioural change techniques	**1- Learn problem-solving skills.** This technique consists in teaching how to analyse factors that influence your behaviour and provide you or help you to develop strategies to reduce or overcome barriers and/or support facilitators (e.g., not eating unhealthy foods when you feel depressed). Strategies include anticipation, self-treatment, resource utilisation, and problem management. Ideally, there should be an initial plan, but this is not a requisite.<br><br>**Example:** Identification and attenuation of environmental barriers (e.g., no gym in the neighbourhood when one wants to exercise) and facilitators (e.g., someone who keeps you company during exercise) to everyday physical activities.<br><br>**2- Goal setting and action planning**. This technique consists in encouraging you to set one or more achievable goals based on your needs and preferences. These goals may be behaviours (e.g., consuming a healthy meal three times a day) or outcomes (e.g., less pain) and can be used as a starting point. The process usually involves the formulation of a detailed action plan, specifying what you would do and at least when and/or where you will do it. It could also include an assessment of your behaviours with your health care provider and a discussion of goals and the writing up of agreed-on action plans, including plans for emergency situations.<br><br>**Examples of goals:** achieving a daily walking distance of 2 km or a weight loss of some kilograms in x months with diet and exercise.
E	Education	**Sharing information**. This form of support consists in sharing information about self-management topics like coping with symptoms, diet, exercise, medication, information about what other people is doing, and information about the disease itself, or about any other relevant aspects that could lead to improved self-management, and ultimately better health. This information can be told or distributed in printed materials like a folder or workbook, or via website or DVD.<br><br>**Examples:** Educational session on healthy eating for people with obesity, provision of a printed leaflet on the importance of foot care in diabetes, or a link to a website with information on chronic obstructive pulmonary disease care.
EB	Emotional-based behavioural change techniques	**1- Stress and/or emotional management.** This technique consists in helping you to understand the role of stress and emotions and teaching you about how to use different coping strategies to manage, for example, stress and painful emotions caused by your disease.<br><br>**Examples:** Mindfulness, exercise, stretching, listening to music, deep breathing, or meditation.<br><br>**2- Coaching and motivational interviewing.** This kind of support helps you to change behaviours by looking at what's important to you, and then offering

		support, taking into account your needs and preferences. One provider (healthcare professional, peer or lay person) is usually your coach. Motivational interviewing and counselling are included, as well as collaborative conversations with a practitioner, helping with motivation and commitment, minimising resistance, and resolving ambivalence to change.<br><br>**Examples:** coaching sessions led by a nurse to ease the transition from hospital to home, or rehabilitation programmes using coaching methods.
G	Group	Two or more patients or caregivers receive a self-management intervention. Group interventions are normally organised for efficiency purposes or to facilitate learning and knowledge exchange among peers (people living with the same health condition). For example, a peer-led education group to enhance physical activity in obese individuals.
IM	Individual or mixed	
MT	Monitoring techniques	**Self-monitoring training and feedback**. Training and encouraging people to recognise, monitor, and record behaviours, symptoms, or clinical data. This process may include regular feedback from a clinician, or a synopsis of information registered in a digital tool to encourage you to continue monitoring your illness and behaviours.<br>**Example:** Showing a patient how to record blood sugar levels, physical activity, or pain.
P	Peers and laypersons or mixed	The intervention is carried out by peers, people who have experienced living with the same disease or by lay people living in your community. Rather than involving just health professionals or educators, the intervention involves one or more peers who take on a role in the teaching and/or providing information about the intervention.<br><br>**Example:** Training and teaching activities provided by peers to guide people with a related health care concern, to adopt a new behaviour that would facilitate healthy outcomes.
R	Remote	When patients (and/or caregivers) are not in the same place as the health care providers. This interaction can take place through different communication means or tools (e.g., telephone, smartphone, Internet).
SD	Shared decision-making	Involving you as an active partner with a provider or multidisciplinary team to encourage and facilitate shared decision-making processes. Within these processes, partners explore different care or treatment options with you, discuss the risks and benefits of each, and reach a joint decision. Shared decision-making is appropriate for any situation where there is more than one reasonable course of action and where no single option is self-evidently best for everyone.<br><br>**Examples:** Discussion of different treatments, procedures, diagnostic tests, or healthy lifestyle behaviours and joint decision on which options are best suited to your needs and preferences.
SS	Social support	Helping you to think through how you could obtain social support from others to help them achieve behavioural or outcome goals. It could also include the actual provision of social support or discussions about social support networks suited to your preferences, needs, disease burden, or additional life burdens.

		Part of this support would include linking you to relevant community services to enhance socialisation and make the most of support mechanisms in the local community.<br><br>**Examples:** Encouraging family members to become involved in helping you to manage your disease or encouraging you to participate in¡ a local exercise group.
CVD: cardiovascular disease; DVD: digital versatile disc.		

Supplementary Table S2: List of critical and important outcomes for T2DM

Critical outcomes	Important outcomes
• HbA1c<br>• Weight management (BMI; waist; weight)<br>• Qol (Qol overall; Psychological distress)<br>• Hypoglycaemia<br>• Blood pressure (SBP; DBP)<br>• Long term complications<br>• Lipid profile (LDL/TG)<br>• Mortality	• Self-efficacy<br>• Self-management (Self-management foot care; Self-management behaviours)<br>• Dietary habits (Dietary habits; Fat consumption)<br>• Knowledge<br>• Physical activity (Physical activity; Steps per day)<br>• Self-monitoring glucose<br>• Adherence<br>• Care satisfaction<br>• Lipid profile (HDL; TC)<br>• Unscheduled visits/hospitalisation

BMI: body mass index; DBP: diastolic blood pressure; Glycated haemoglobin: glycosylated haemoglobin; HDL: high-density lipoprotein; LDL: low-density lipoprotein; QoL: quality of life; SBP: systolic blood pressure; T2DM: type 2 diabetes mellitus; TC: total cholesterol; TG: triglycerides

Supplementary Figure S1: PRISMA flowchart

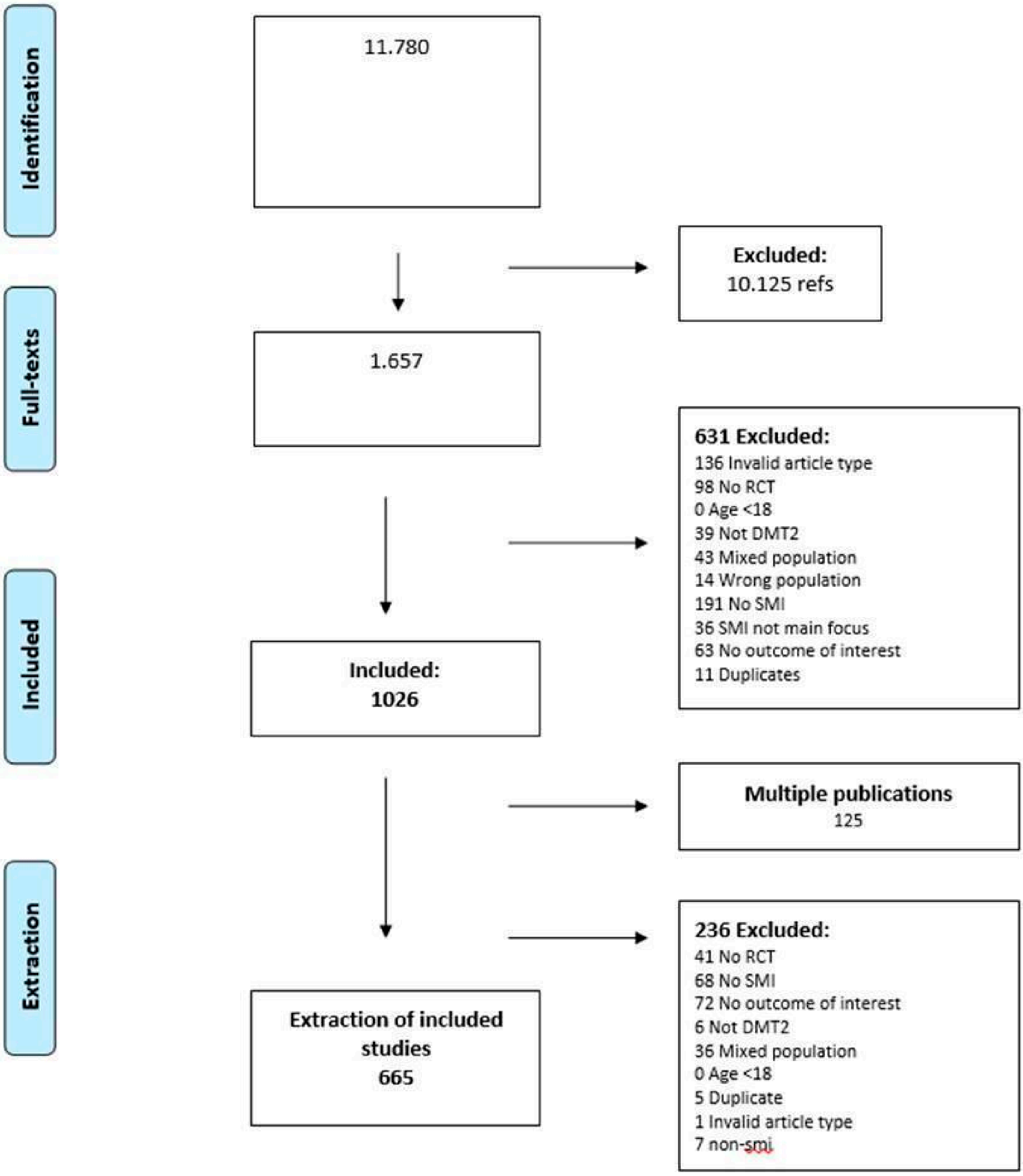

Figure Diabetes: Preferred Reporting Items for Systematic Reviews and Meta-Analyses (PRISMA) flow chart

Supplementary Link S1. Evidence to Decision Framework for all SMIs

Supplementary Figure S2: Meta-regression of all SMI vs UC for the outcome Glycated haemoglobin reduction

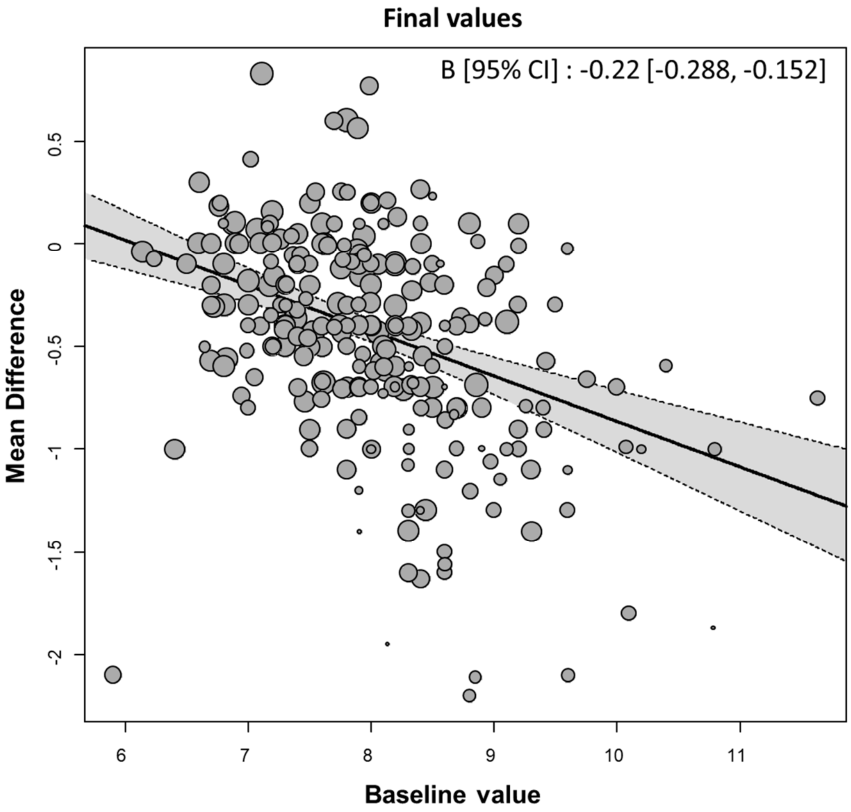

Supplementary Table S3: Intensity subgroup analysis

Outcome	Sub.group (Intensity high >10h / low<10 h)	N° studies	Random.95.CI	tau2	I^2
HBA1C	Low	368	TE: -0.37 95% CI: [0.32 , 0.42]	0,41	0,99
	High	40	TE: **-0.42** 95% CI: [0.28 , 0.57]	0,4	0,94
Diastolic pressure	Low	167	TE: -1.13 95% CI: [0.74 , 1.51]	1,87	0,87
	High	20	TE: **-1.45** 95% CI: [0.83 , 2.06]	0	0
Systolic pressure	Low	185	TE: **-2.08** 95% CI: [1.5 , 2.66]	2,92	0,81
	High	23	TE: -1.51 95% CI: [-0.03 , 3.04]	2,62	0,53
BMI	Low	199	TE: **-0.28** 95% CI: [0.14 , 0.41]	0,71	0,91
	High	19	TE: -0.02 95% CI: [-0.26 , 0.31]	0,39	0,54
Hypoglycaemia	Low	9	TE: -0.14 95% CI: [-0.38 , 0.65]	0,69	0,95
	High	1	TE: **-0.84** 95% CI: [0.02 , 1.67]	NA	NA
Psychological distress	Low	28	TE: -0.11 95% CI: [0.02 , 0.21]	0,18	0,52
	High	2	TE: **-0.29** 95% CI: [0.03 , 0.56]	0,17	0,78
Quality of life	Low	67	TE: 0.23 95% CI: [-0.36 , -0.1]	0,5	0,86
	High	6	TE: **0.33** 95% CI: [-0.85 , 0.19]	0,63	0,88
Waist size	Low	71	TE: **-1.56** 95% CI: [1 , 2.12]	1,68	0,64
	High	5	TE: -0.32 95% CI: [-0.19 , 0.84]	0,25	0,03
Weight	Low	115	TE: **-1.02** 95% CI: [0.59 , 1.45]	1,64	0,75
	High	6	TE: -0.97 95% CI: [-0.01 , 1.94]	0	0
LDL	Low	9	TE: -0.14 95% CI: [-0.38 , 0.65]	0,69	0,95
	High	1	TE: **-0.84** 95% CI: [0.02 , 1.67]	NA	NA
Triglycerides	Low	137	TE: -0.09 95% CI: [0.02 , 0.17]	0,35	0,89
	High	13	TE: **-0.26** 95% CI: [0 , 0.52]	0,33	0,58

Supplementary Figure S3: Subgroup analysis per component

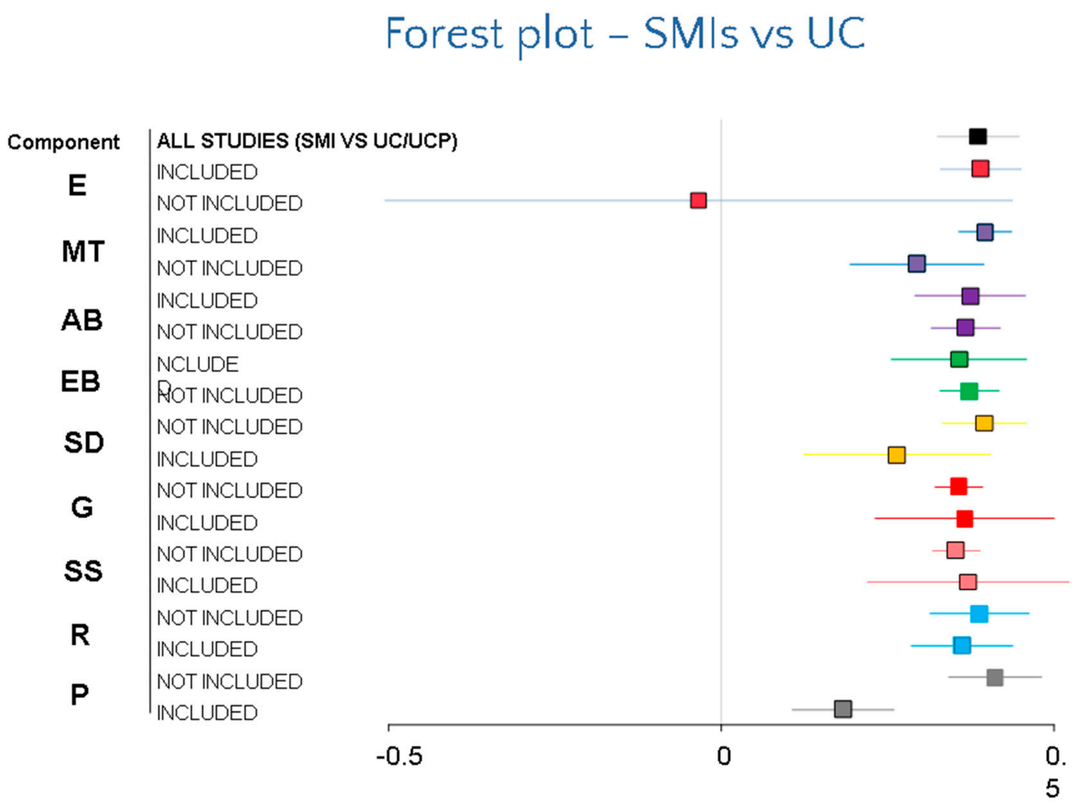

AB=Action-based behavioural change techniques; E=Education; EB=Emotional-based behavioural change techniques; G=Group; IM=Individual or mixed; MT=Monitoring techniques; P=Peers and laypersons or mixed; R=Remote; SD=Shared decision-making; SS=Social support

Supplementary Figure S4: Heat plots graphs

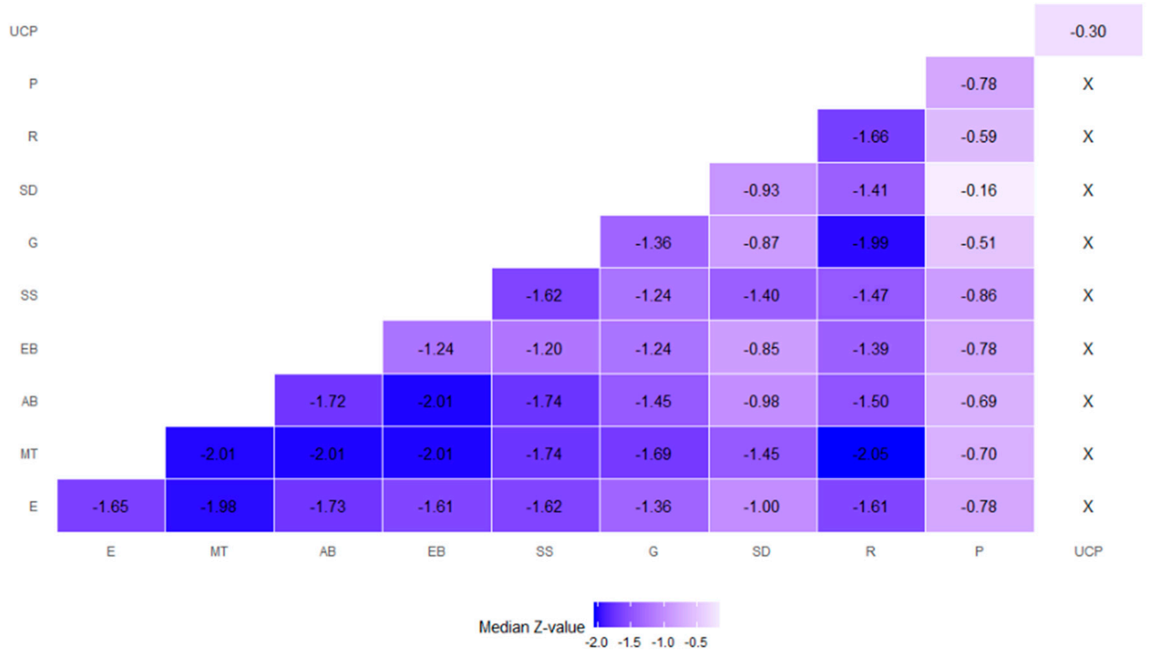

AB=Action-based behavioural change techniques; E=Education; EB=Emotional-based behavioural change techniques; G=Group; IM=Individual or mixed; MT=Monitoring techniques; P=Peers and laypersons or mixed; R=Remote; SD=Shared decision-making; SS=Social support

Diagonal elements refer to components, while non-diagonal elements refer to the combination of components. Each element summarises the z-value of the interventions that include the corresponding component (combination), using the median as a summary measure. Also, the intensity of the colour is proportional to the magnitude of the statistical evidence. Thus, dark blue colour indicates strong statistical evidence that the underlying component (combination) performs better than UC. For example, for the outcome of HbA1c, most squares are purple, indicating that the components are working. Monitor techniques have the largest z-value indicating strong statistical evidence that monitor techniques perform better than UC.

Supplementary Link S2. Evidence to Decision Frameworks for the selected interventions

Supplementary Table S4. Multiple comparison summary of judgements table for selected SMIs

Selected SMIs	Balance of effects	Certainty of evidence	Resources required	Cost-effectiveness	Equity	Acceptability	Feasibility
Monitoring techniques led by peers with or without professionals delivered in groups	Probably favours the intervention	⊕⊕◯◯Low	Moderate costs	Probably favours the intervention	Probably increased	Probably yes	Probably yes
Emotional-based behavioural techniques led by peers with or without professionals, delivered remotely	Probably favours the intervention	⊕⊕◯◯Low	Moderate costs	Does not favour either	Probably increased	Probably yes	Probably yes
Monitoring, action-based behavioural techniques, and shared decision-making delivered in groups	Probably favours the intervention	⊕⊕◯◯Low	Moderate costs	Does not favour either	Probably increased	Probably yes	Probably yes
Monitoring, action-based and emotional-based behavioural techniques and social support led by peers with or without professionals delivered remotely	Probably favours the intervention	⊕⊕◯◯Low	Moderate costs	Does not favour either	Probably increased	Probably yes	Probably yes
Emotional-based behavioural techniques and social support delivered in groups	Probably favours the intervention	⊕⊕◯◯Low	Moderate costs	Does not favour either	Probably increased	Probably yes	Probably yes
Action-based behavioural techniques, social support led by peers and professionals	Probably favours the intervention	⊕⊕◯◯Low	Moderate costs	Does not favour either	Probably increased	Probably yes	Probably yes
Education delivered in groups and remotely	Probably favours the intervention	⊕⊕◯◯Low	Negligible costs and savings	Does not favour either	Probably increased	Probably yes	Probably yes
Monitoring techniques and social support delivered remotely	Probably favours the intervention	⊕⊕◯◯Low	Don't know	Does not favour either	Probably increased	Probably yes	Probably yes
Monitoring and action-based behavioural techniques, shared decision making and social support, delivered in groups	Probably favours the intervention	⊕⊕◯◯Low	Moderate costs	Does not favour either	Probably increased	Probably yes	Probably yes
Monitoring and emotional-based behavioural techniques delivered remotely	Probably favours the intervention	⊕⊕◯◯Low	Moderate costs	Does not favour either	Probably increased	Probably yes	Probably yes

Supplementary Table S5: Implementation considerations per component.

Component	Strategies included
SMI	**Healthcare providers' level:** it is important to adapt the advice, communication or intervention to a patient's personal situation and level of knowledge; to have adequate communication skills (for example, show empathy, provide understandable information, ask questions);<br><br>**Patients' level:** patient's motivation to engage in self-management; patient's attitude towards self-management (for example, beliefs about the importance of self-management for health, beliefs about the usefulness of certain self-management tasks).<br><br>**Interaction level:** patients' preference regarding their own role in their treatment (for example, the extent to which a patient wants to be involved in shared decision-making, extent to which a patient expects or wants professional involvement in the daily management of their disease).
Education	**Healthcare providers' level:** it is important to adapt the advice, communication or intervention to a patient's personal situation and level of knowledge; to have adequate communication skills (for example, show empathy, provide understandable information, ask questions);<br><br>**Patients' level:** patient's motivation to engage in self-management; patient's cognitive and behavioural skills to self-management.
Monitoring Techniques	**Healthcare providers' level:** it is important to adapt the advice, communication or intervention to a patient's personal situation and level of knowledge; to be aware of your attitude toward the patients' knowledge and personal beliefs.<br><br>**Patients' level:** patient's motivation to engage in self-management; patient's attitude towards self-management.<br><br>**Interaction level:** patients' preference regarding their own role in their treatment (for example, the extent to which a patient wants to be involved in shared decision-making, extent to which a patient expects or wants professional involvement in the daily management of their disease)
Action-based behavioural change techniques	**Healthcare providers' level:** it is important to adapt the advice, communication or intervention to a patient's personal situation and level of knowledge;<br><br>**Patients' level:** patient's motivation to engage in self-management; patient's attitude towards self-management (for example, beliefs about the importance of self-management for health, beliefs about the usefulness of certain self-management tasks).<br><br>**Interaction level:** patients' preference regarding their own role in their treatment (for example, the extent to which a patient wants to be involved in shared decision-making, extent to which a patient expects or wants professional involvement in the daily management of their disease)

Emotional-based change techniques	**Healthcare providers' level:** it is important to adapt the advice, communication or intervention to a patient's personal situation and level of knowledge; to have adequate communication skills (for example, show empathy, provide understandable information, ask questions);<br><br>**Patients' level:** patient's motivation to engage in self-management;<br><br>**Interaction level:** patients' preference regarding their own role in their treatment (for example, the extent to which a patient wants to be involved in shared decision-making, extent to which a patient expects or wants professional involvement in the daily management of their disease).
Social Support	**Healthcare providers' level:** it is important to adapt the advice, communication or intervention to a patient's personal situation and level of knowledge; to have expertise in supporting self-management for chronic diseases;<br><br>**Patients' level:** (perceived) available support from family and friends; peer support and interaction
Shared decision making	**Healthcare providers' level:** it is important to adapt the advice, communication or intervention to a patient's personal situation and level of knowledge; to have adequate communication skills (for example, show empathy, provide understandable information, ask questions);<br><br>**Patients' level:** patient's motivation to engage in self-management; patient's knowledge and personal beliefs about the disease and its treatment;<br><br>I**nteraction level:** patients' preference regarding their own role in their treatment (for example, the extent to which a patient wants to be involved in shared decision-making, extent to which a patient expects or wants professional involvement in the daily management of their disease).
Groups and/or Peers	**Healthcare providers' level:** it is important to adapt the advice, communication or intervention to a patient's personal situation and level of knowledge; to have adequate communication skills (for example, show empathy, provide understandable information, ask questions); to have adequate skills in monitoring group interactions.<br><br>**Patients' level:** patients' motivation to engage in self-management; to know and adapt to the cultural background and/or language of the patient; patients' attitude towards self-management (for example, beliefs about the importance of self-management for health, beliefs about the usefulness of certain self-management tasks).<br><br>**Interaction level:** to know patients' preference regarding their own role in their treatment (for example, the extent to which a patient wants to be involved in shared decision-making, extent to which a patient expects or wants professional involvement in the daily management of their disease).<br><br>**Other contextual factors:** to have education and continuous support of peer providers.
Remote	**Healthcare providers' level:** it is important to adapt the advice, communication or intervention to a patient's personal situation and level of knowledge; to have adequate communication skills (for example, show empathy, provide understandable information, ask questions);

	**Patients' level:** patient's motivation to engage in self-management.<br><br>**System level:** the availability of a suitable infrastructure for self-management support
